# The Colorimetric Isothermal Multiple-Self-Matching-Initiated Amplification Using Cresol Red for Rapid and Sensitive Detection of Porcine Circovirus 3

**DOI:** 10.3389/fvets.2020.00407

**Published:** 2020-08-04

**Authors:** Hongchao Gou, Zhibiao Bian, Rujian Cai, Zhiyong Jiang, Shuai Song, Yan Li, Pinpin Chu, Dongxia Yang, Ying-An Zang, Chunling Li

**Affiliations:** ^1^Institute of Animal Health, Guangdong Academy of Agricultural Sciences, Guangzhou, China; ^2^Guangdong Provincial Key Laboratory of Livestock Disease Prevention, Guangzhou, China; ^3^Guangdong Open Laboratory of Veterinary Public Health, Guangzhou, China; ^4^Scientific Observation and Experiment Station of Veterinary Drugs and Diagnostic Techniques of Guangdong Province, Guangzhou, China; ^5^College of Animal Science and Technology, Zhongkai University of Agriculture and Engineering, Guangzhou, China

**Keywords:** PCV3, isothermal multiple-self-matching-initiated amplification (IMSA), detection, colorimetric assay, cresol red

## Abstract

In 2016, a novel porcine circovirus (PCV), PCV3, was identified in USA. Subsequently, it was proved to be also epidemic in China, Poland, and Korea. To analyze and control the epidemic situation of PCV3, it is necessary to establish accurate and high-throughput detection methods. In this study, the colorimetric isothermal multiple-self-matching-initiated amplification (IMSA) using cresol red was developed to detect PCV3 for the first time. The reaction can be easily performed by incubating the tube at 63°C for 60 min. By the addition of pH-sensitive indicator dye cresol red, the initial color of the reaction mixture is red. When PCV3 capsid gene DNA was positive in the sample, the color of the reaction mixture changed from red to yellow after the isothermal incubation at 63°C, while the negative control maintained the red color. The colorimetric IMSA displayed good specificity in detecting PCV3, PCV2, and PCV1 and 4 porcine DNA pathogens. Moreover, it has a low and repeatable detection limit of 10 copies, which is consistent with TaqMan-based qPCR, but 10 times more sensitive than PCR. In diagnosing 128 clinical specimens, it not only showed 100% agreement with qPCR but also detected 15 positive results more than PCR. The colorimetric IMSA we offered might be a good choice for PCV3 epidemiological investigation and point-of-care testing.

## Introduction

Porcine circovirus (PCV) is a non-enveloped, circular single-stranded DNA virus, belonging to the genus *Circovirus* within the family *Circoviridae*. PCV1 and PCV2 were reported to be the only two members of PCV for a long time (1). PCV1 was considered non-pathogenic to pigs. However, PCV2 was known to be connected with clinical features including post-weaning multisystemic wasting syndrome (PMWS), porcine dermatitis and nephropathy syndrome (PDNS), porcine respiratory disease complex (PRDC), proliferative and necrotizing pneumonia (PNP), and reproductive disorders (2, 3). PCV2 has caused severe economic losses to the swine industry because of its circulation worldwide (4).

In 2016, a novel porcine circovirus, PCV3, was identified in USA. Similar symptoms with pigs infected by PCV2, such as PDNS and reproductive disorders, also appear in pigs infected by PCV3 (5, 6). Successive studies proved it was epidemic in China, Poland, and Korea (7–10). Considering this, it is necessary to develop a rapid and simple assay for PCV3 detection. Although PCR-based methods have been utilized to detect PCV3, it is not easy to be performed onsite, or laboratories lack expensive thermal cycling equipment and skilled operators (7, 11). Therefore, isothermal amplification methods are suggested to be a suitable choice for point-of-need diagnostics. To meet this demand, recombinase polymerase amplification (RPA) assay for rapid detection of PCV3 has been described (12). However, at least two kinds of enzymes are needed to start the RPA reaction. The expensive reaction reagent may limit its wide application in rural areas.

Compared with RPA, loop-mediated isothermal amplification (LAMP) or isothermal multiple-self-matching-initiated amplification (IMSA) only utilizes strand-displacing DNA polymerase to accomplish robust DNA synthesis (13, 14). This makes them to be good choice for low-cost isothermal methods. What is more, IMSA proved to be more sensitive than LAMP in the previous study (13, 15). Recently, rapid, sensitive, and visual detection assay was achieved by the addition of pH-sensitive indicator dyes. To develop a rapid, convenient, low-cost method suitable for rural areas, the colorimetric IMSA using cresol red was utilized to detect PCV3 in our study. This method showed good potential on rapid examination of clinical samples suspected to be infected by PCV3.

## Materials and Methods

### Ethics Statement

All animal experiments were reviewed and approved by the ethical and ethics commission (Institute of Animal Health, Guangdong Academy of Agricultural Sciences, China). The license number was SYXK (Yue) 2011–0116. Moreover, samples collecting treatment in this study were performed in accordance with national and local laws and guidelines.

### Virus, Bacteria, and Cells

PCV2 isolate HN6 (GenBank no: KM035762.1), PCV1, pseudorabies virus (PRV) GD-WH strain (GenBank no: KT936468.1), Haemophilus parasuis (HPS) serotype 5, *Streptococcus suis* (SS) serotype 2, and *Actinobacillus pleuropneumoniae* (APP) Serovar 1 were preserved in our laboratory. They were used to evaluate the specificity of the colorimetric IMSA.

### Clinical Samples and Animals

In Guangdong province, a total number of 128 clinical samples were collected from pigs suspected to be infected with PCV3. The tissues include blood, tonsil, lymph gland, lung, kidney, and brain. In addition, 15 blood samples of specific-pathogen-free (SPF) pigs (5 months old), from the Laboratory Animal Center of Southern Medical University, China, were collected. All samples were stored at −80°C until DNA extraction.

### DNA Extraction

According to the instructions of the manufacturer, viral DNA in supernatant of cell culture or clinical samples was purified by using the HiPure Viral RNA/DNA Kit (Magen, China). Finally, viral DNA was dissolved in 50 μL nuclease-free water and stored at −80°C. Bacterial DNA was extracted by using the HiPure Bacterial DNA Kit (Magen, China) according to the manufacturer's protocol.

### PCR

According to the previously reported methods, PCR was performed in a 25-μL reaction mixture (7). The mixture contained 0.3 μM of each primer, 1 × Premix Tag (Takara Biotechnology, China) and 2 μL template DNA. The PCR program was as follows: 94°C for 5 min; 35 cycles at 94°C for 30 s, 55°C for 30 s, and 72°C for 1 min, and a final extension at 72°C for 10 min. The products were analyzed on 2% agarose gel.

### TaqMan-Based qPCR

TaqMan-based qPCR for PCV3 genome identification was carried out as is previously reported (6). The 25-μL reaction mixture contained 0.4 μM of each primer and probe, 1 × qPCR Probe Master Mix (Vazyme, China), and 2 μL template DNA. The reaction program was set as follows: 95°C for 3 min, followed by 40 cycles at 95°C for 10 s and 60°C for 60 s. FAM fluorescence signals were obtained at the end of each annealing step by the real-time PCR detection system (Roche Light Cycler 480 II, Switzerland). Results with a cycle threshold (Ct) value of <40 were considered positive, while results with no Ct value within 40 cycles were considered negative.

### Plasmid Construction

As in the report previously described, partial sequences of capsid gene of PCV3 were amplified by the PCR method from the clinical samples (7). The PCR product was purified by using the Cycle Pure Kit according to the instructions of the manufacturer (Omega, USA). Then, the purified fragment was cloned into pMDTM19-T Vector (TaKaRa Biotechnology, China) and the pMD19T-capsid plasmid was constructed. After the plasmid was transformed into DH5α competent cells, the plasmid DNA was extracted by using the Plasmid Mini Kit I (Omega, USA).

### IMSA Primer Design

The conserved region of the capsid gene was determined by alignment of PCV3 strains indexed in the GenBank (accession no: MF589105.1, MF589107.1, MF769811.1, MF769807.1, MF084994.1, KX778720.1, KX898030.1, MG310152.1, MF079254.1, and MG250187.1). According to the principle of IMSA assay, capsid gene sequences of PCV3-US/MO2015 strain (accession no: KX778720.1) were input for IMSA primer design by using the software Primer Premier 5.0 (15). Among multiple sets of primers, the primers targeting to the conserved regions of the capsid gene were selected for subsequent analysis. IMSA primers are listed in Table 1.

**Table 1:** Primers of the colorimetric IMSA assay for PCV3.

**Primer name**	**Sequences 5^**′**^-3^**′**^**	**Genome position^**a**^**
DsF	CCCACCCCATGGCTCAACA-CCGGGACATAAATGCTCCAA	SteR: 1465–1483 F3: 1411–1430
DsR	TGGTTTCGGGGTGAAGTAACGG-CCACAAACACTTGGCTCCA	SteF: 1541–1562 R3: 1623–1641
FIT (F1c+F2)	CTCACCCAGGACAAAGCCTCTT-CATTGAACGGTGGGGTCAT	Tc: 1497–1518 F2: 1443–1461
RIT (B1c+B2)	AAGAGGCTTTGTCCTGGGTGAG-AGACGACCCTTATGCGGAA	T: 1497–1518 R2: 1604–1622
SteR	CCCACCCCATGGCTCAACA	1465–1483
SteF	TGGTTTCGGGGTGAAGTAACGG	1541–1562

a *The genome of PCV3-US/MO2015 strain (accession no: KX778720.1)*.

### Colorimetric IMSA

As the previous study described, Tris–HCl and betaine was not included in the colorimetric assay (16). To establish this method, the reaction mixture containing 10 mM (NH4)_2_SO_4_, 10 mM KCl, 8 mM MgSO_4_, 0.1% v/v Tween-20, 16 mM cresol red, 0.8 mM dNTPs, and 8 U Bst WarmStart DNA Polymerase (New England Bio-labs, USA) was prepared, and the pH of the mixture was adjusted to 9.0 by using 100 mM KOH. To perform the IMSA reaction, primers' concentration was optimized and determined as 1.6 μM SteF/SteR, 0.8 μM FIT/RIT, and 0.2 μM DsF/DsR. Then, the reaction tube was incubated in a thermostatic device (HB-202, BIOER TECHNOLOGY, China) at 63°C for 60 min. The results can be directly judged with the naked eyes. The yellow color indicated the positive results, while the red color indicated the negative results. In each colorimetric IMSA test, 3 observers were invited to identify colors and read out the results. For each result, every observer read it out 3 times.

### Specificity Analysis

DNA extracted from PCV2, PCV1, PRV GD-WH strain, HPS, SS, and APP were used to evaluate the specificity of the colorimetric IMSA.

### Sensitivity Analysis

Ten-fold serial dilutions of pMD19T-capsid plasmid DNA (10^5^, 10^4^, 10^3^, 10^2^, 10^1^, 10^0^, and 10^−1^ copies) were used to determine the detection limit of the colorimetric IMSA. The negative control was conducted at the same time. The detection limit of the colorimetric IMSA was compared with qPCR and PCR.

### Reproducibility Analysis

To analyze the reproducibility of the colorimetric IMSA, ten-fold serial dilutions of pMD19T-capsid plasmid DNA (10^5^, 10^4^, 10^3^, 10^2^, 10^1^, 10^0^, and 10^−1^ copies) and the negative control were detected for 3 different times.

### Evaluation of Clinical Application

A total number of 128 suspected clinical samples and 15 blood samples from SPF pigs were used for DNA extraction and colorimetric IMSA detection. To evaluate the test accuracy of the colorimetric IMSA, its results were identified by TaqMan-based qPCR and PCR.

## Results

### Establishment of the Colorimetric IMSA

**Figure 1:**
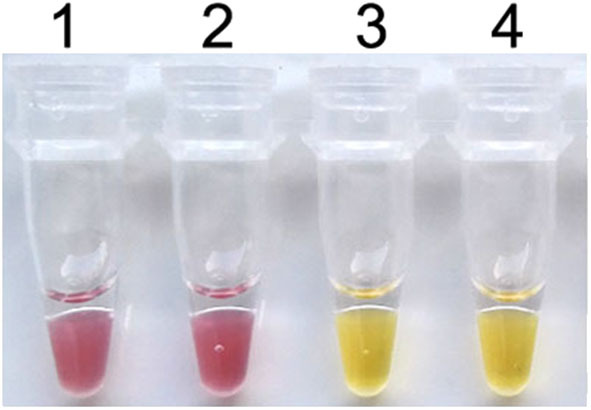
Establishment of the colorimetric IMSA assay for PCV3. Tubes 1–2, negative control. Tubes 3–4, 10^5^ copies of PMD19T-capsid plasmid DNA.

To establish the colorimetric IMSA assay, Tris–HCl was removed from the reaction mixture but the cresol red was added. After 60 min of incubation at 63°C, the color of the reaction mixture changed to yellow when the plasmid containing partial sequences of the PCV3 capsid gene was used as DNA template, while the negative control keeps the red color (Figure 1). So, we judged the positive result of the colorimetric IMSA by observing the yellow color of the reaction tube.

### Specificity of the Colorimetric IMSA

**Figure 2:**
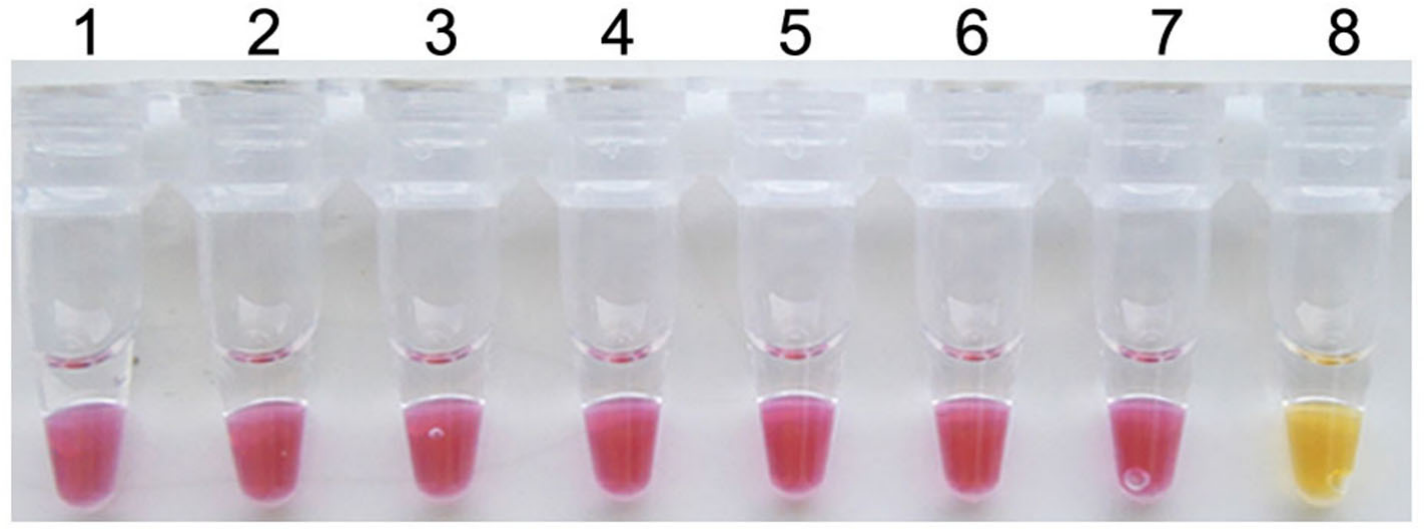
Specificity of the colorimetric IMSA assay for PCV3. Tubes 1–6, DNA of PCV2, PCV1, PRV GD-WH strain, HPS, SS, and APP. Tube 7, negative control. Tube 8, 10^5^ copies of PMD19T-capsid plasmid DNA.

To evaluate the specificity of the colorimetric IMSA, pathogens inducing similar clinical syndromes with PCV3, such as PCV2, PCV1, PRV GD-WH strain, HPS, SS, and APP, were used for DNA extraction. For multiple viral or bacterial DNAs, only the plasmid containing partial sequences of the PCV3 capsid gene could start the reaction and the reaction mixture displayed the yellow color (Figure 2). This manifested the good specificity of the colorimetric IMSA.

### Sensitivity of the Colorimetric IMSA

**Figure 3:**
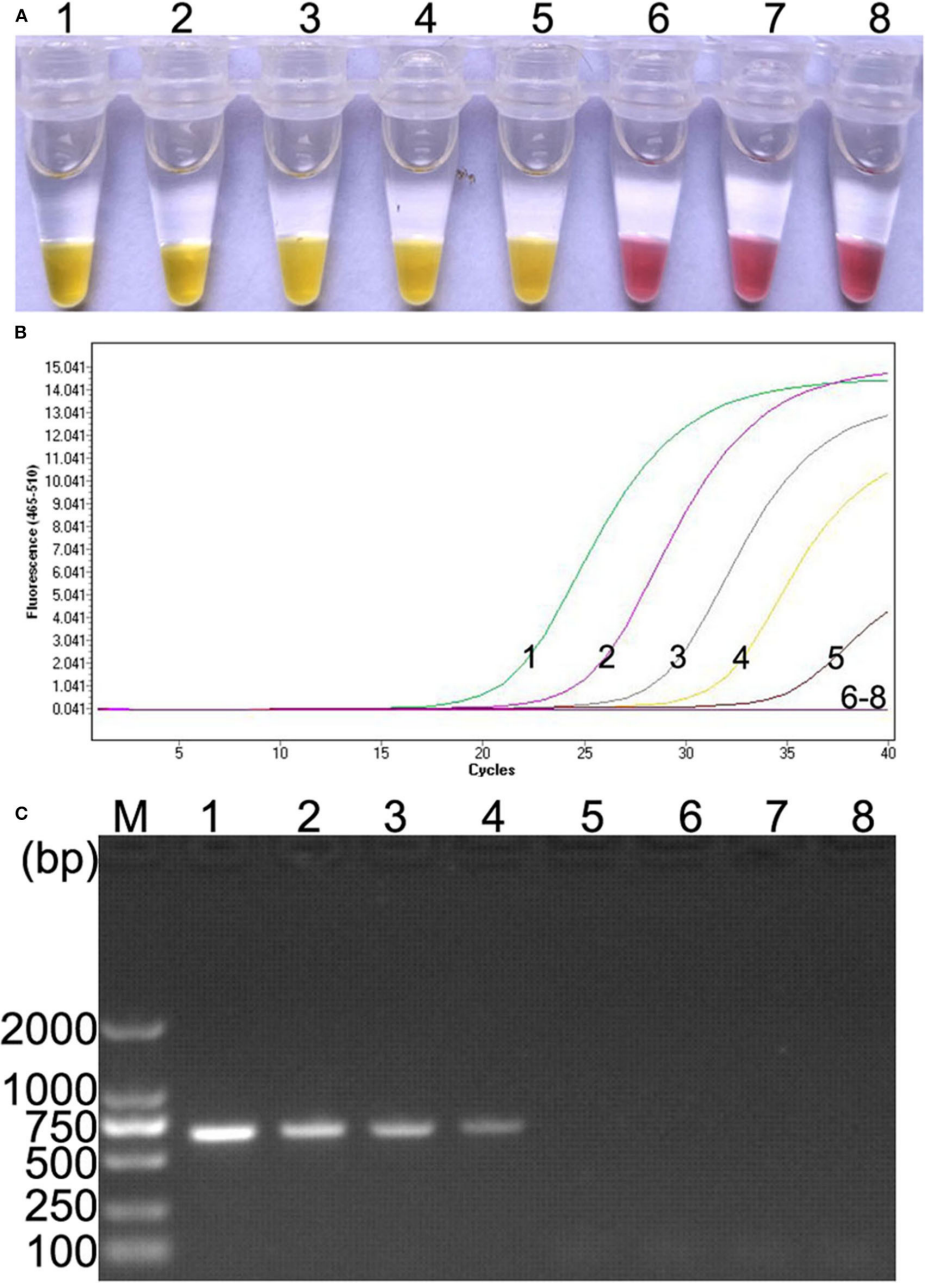
Comparison of sensitivity of the colorimetric IMSA with qPCR and PCR. **(A)** Sensitivity of the colorimetric IMSA. **(B)** Sensitivity of qPCR. **(C)** Sensitivity of PCR analyzed by agarose gel electrophoresis. Tubes or lanes 1–7, ten-fold serially diluted PMD19T-capsid plasmid DNA (10^5^, 10^4^, 10^3^, 10^2^, 10^1^, 10^0^, and 10^−1^ copies). Tube or lane 8: negative control. Lane M, 2,000-bp DNA marker.

Ten-fold serial dilutions of plasmid DNA (10^5^, 10^4^, 10^3^, 10^2^, 10^1^, 10^0^, and 10^−1^ copies) were, respectively, used as DNA template to determine the detection limit of the colorimetric IMSA. Moreover, the results were compared with qPCR and PCR. In this study, as low as 10 copies of plasmid DNA can be detected by the colorimetric IMSA and qPCR, while the detection limit of PCR was 100 copies, which was 10 times lower than the colorimetric IMSA and qPCR (Figure 3).

### Reproducibility of the Colorimetric IMSA

**Figure 4:**
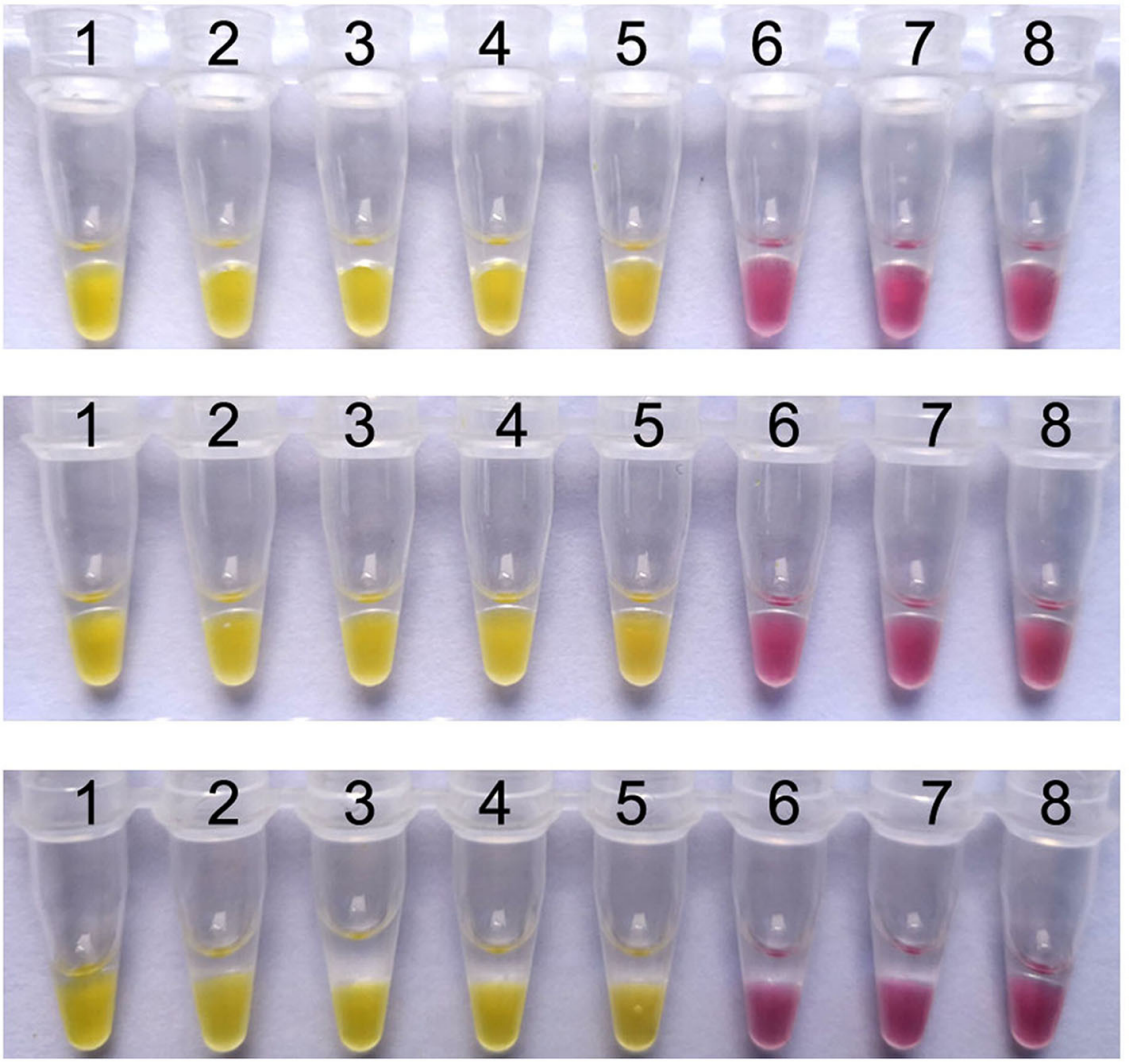
Reproducibility analysis of the colorimetric IMSA. Tubes 1–7, ten-fold serially diluted PMD19T-capsid plasmid DNA (10^5^, 10^4^, 10^3^, 10^2^, 10^1^, 10^0^, and 10^−1^ copies). Tube 8: negative control.

When the colorimetric IMSA was evaluated by detecting ten-fold serial dilutions of pMD19T-capsid plasmid DNA (10^5^, 10^4^, 10^3^, 10^2^, 10^1^, 10^0^, and 10^−1^ copies) and the negative control for 3 different times, the same results were obtained for each dilution sample every time (Figure 4). Hence, the colorimetric IMSA showed stable detection reproducibility for samples of different concentrations.

### Evaluation of Clinical Application

Among 128 clinical samples, 35 positive results were detected by the PCR method. The positive rate was 27.3% (35/128). In addition to these 35 positive results, the colorimetric IMSA showed 15 positive results more than PCR, which were also positive for TaqMan-based qPCR (Table 2). The positive rate was 39.1% (50/128). To analyze the accuracy of IMSA in testing negative animals, 15 blood tissues sampled from SPF pigs were detected by using the colorimetric IMSA, qPCR, and PCR. Fifteen negative results were obtained for these 3 methods (data not shown).

**Table 2:** Detection of PCV3 in clinical samples by the colorimetric IMSA qPCR and PCR.

**Tissues**	**Number**	**Positive by qPCR**	**Positive by PCR**	**Positive by IMSA**
Blood	36	15	8	15
Tonsil	15	8	6	8
Lymph gland	12	5	4	5
Lung	26	6	5	6
Kidney	22	11	9	11
Brain	17	5	3	5
Total	128	50	35	50
Positive rates^a^ (%)	/	39.1%	27.3%	39.1%

a *Detection rate was defined by no. of positive samples/no. of total samples (%)*.

## Discussion

PCV2, the main inducer of porcine circovirus-associated diseases (PCAD), severely damaged efficiency of swine production worldwide (1). PCV3, which was firstly identified in PCV2-negative pigs in 2016, causes cardiac pathology and multisystemic inflammation. In addition, PCV2-like syndromes including PDNS and reproductive failure are also associated with PCV3 infection (6). To analyze and control the epidemic situation of PCV3, it is necessary to establish accurate and high-throughput detection methods. Herein, a simple and rapid colorimetric IMSA using cresol red was developed to detect PCV3 for the first time.

In our study, the IMSA primers were designed according to the conserved region of the PCV3 capsid gene, which was the usual detective marker of PCR-based methods (7, 11). The primer design work was not as complicated as those of LAMP, because the IMSA primers consisted of seven basic primers specifically recognizing distinct regions of the target, which can be easily designed by using the software Primer Premier 5.0 (15). The results of colorimetric IMSA can be visually judged within 60 min, which was at least 60 min shorter than qPCR or PCR methods. However, the detection limit of the colorimetric IMSA is as low as 10 copies, which is consistent with qPCR and even 10 times more sensitive than PCR previously reported (7, 11). Moreover, the reproducibility analysis shows that the detection accuracy of the colorimetric IMSA is very stable for samples of different concentrations. In the whole detection process, the colorimetric IMSA only needs simple isothermal equipment to perform the reaction, but results of PCR or qPCR need to be analyzed by agarose gel electrophoresis or sophisticated thermal cycling apparatus. This makes it very suitable for point-of-care tests using the colorimetric IMSA assay. Compared with the LAMP assay using hydroxynaphthol blue for PCV3 detection, the color change from red to yellow of the reaction tube in this study is more easily to be distinguished with the naked eye than that of the color change from purple to sky blue (17). Hence, it is very convenient for the observers to read out and judge the results of the colorimetric IMSA.

When the colorimetric IMSA are used to detect clinical samples, all IMSA-positive samples can be exactly determined by TaqMan-based qPCR, which was proved to be an accurate method for PCV3 DNA detection. Moreover, the colorimetric IMSA displayed no false-positive results for detection of blood samples from SPF pigs. This further manifests its precise test ability. In our results, some IMSA-positive samples were negative for PCR detection. This may be attributed to the more sensitive detection limit of the colorimetric IMSA and qPCR than PCR.

In recent years, PCV3 was continuously identified in China and further surveillance studies showed that PCV3 are epidemic in a number of pig farms in many provinces (7, 10). In our study, the positive detection rate of the suspected clinical samples by colorimetric IMSA and qPCR is 39.1% (50/128), which was higher than the data in the previous study (11, 12). Our data offers new evidence that PCV3 was epidemic in sick pigs in China. As an emerging virus in 2016, some reports have manifested its connection with PDNS. Pathological lesions and PCV3-specific antigens are detected in various tissues and organs, including the blood, lung, heart, kidney, lymph nodes, and spleen (6, 18). Our results also showed that PCV3 is widely distributed in multiple tissues of the suspected infection pigs. What is more, we find that PCV3 DNA can be detected in the brain tissues of some sick pigs. This is consistent with the findings in some recent reports (19, 20).

In summary, the IMSA assay using cresol red was used for a rapid and sensitive detection of PCV3 for the first time. Not only was this method easy to be performed, but also its results were clearly distinguished with naked eyes. It is likely that the simple method was widely applied in the PCV3 detection in laboratories or rural areas.

## Data Availability Statement

The datasets generated for this study are available on request to the corresponding author.

## Ethics Statement

This animal study was reviewed and approved by the ethical and ethics commission (Institute of Animal Health, Guangdong Academy of Agricultural Sciences, China). The license number was SYXK (Yue) 2011–0116. Moreover, sample collecting treatment in this study were performed in accordance with national and local laws and guidelines.

## Author Contributions

HG carried out the experiment design and drafted the manuscript. PC and DY prepared materials for the experiments. ZB participated in the experiments. SS, ZJ, and YL participated in the analysis of the data. CL, Y-AZ, and RC conceived the study. All authors read and approved the final manuscript.

## Conflict of Interest

The authors declare that the research was conducted in the absence of any commercial or financial relationships that could be construed as a potential conflict of interest.
